# Milk-Fat Intake and Differences in Abdominal Adiposity and BMI: Evidence Based on 13,544 Randomly-Selected Adults

**DOI:** 10.3390/nu13061832

**Published:** 2021-05-27

**Authors:** Klarissa R. Wilkinson, Larry A. Tucker, Lance E. Davidson, Bruce W. Bailey

**Affiliations:** College of Life Sciences, Brigham Young University, Provo, UT 84602, USA; klarissa.wilkinson@gmail.com (K.R.W.); lance.davidson@byu.edu (L.E.D.); bruce_bailey@byu.edu (B.W.B.)

**Keywords:** overweight, obesity, sagittal diameter, waist circumference, dairy, diet

## Abstract

The primary purpose of this investigation was to evaluate the relationship between milk-fat intake and obesity, particularly abdominal obesity, in 13,544 U.S. adults. A lesser objective was to measure the degree to which the association was influenced by multiple potential confounding variables. This cross-sectional study used data from the 2011–2016 National Health and Nutrition Examination Survey (NHANES). Quantity of milk-fat regularly consumed was the exposure variable. Sagittal abdominal diameter (SAD), a measure of abdominal obesity, and body mass index (BMI) were the outcome variables. Sagittal abdominal diameter is a strong predictor of visceral abdominal fat, when measured by computed tomography, and has been shown to predict cardiometabolic disorders better than BMI. After controlling for age, race, gender, physical activity, leisure computer use and gaming, alcohol habits, and cigarette use, significantly lower BMIs were associated with consistent non-fat and full-fat milk consumption (F = 4.1, *p* = 0.0063). A significantly lower SAD was associated only with regular consumption of non-fat milk (F = 5.0, *p* = 0.0019). No significant differences were detected between the other milk-fat groups or milk abstainers. In this nationally representative sample, only 19.6% of adults regularly consumed low-fat milk. In conclusion, consistent non-fat milk intake was predictive of lower levels of abdominal adiposity compared to consumption of higher levels of milk-fat.

## 1. Introduction

Obesity is a disease characterized by a body mass index (BMI) of ≥ 30 kg/m^2^ [1]. Over the past several decades, the incidence of obesity in the United States has increased considerably. Results from the 2017–2018 National Health and Nutrition Examination Survey (NHANES) indicate that 42.4% of U.S. adults have obesity, and 9.2% have severe obesity [2]. This growing trend brings with it many concerns. An evaluation of the health outcomes associated with obesity indicates that as BMI increases, incidence of some cancers, type 2 diabetes, heart disease, and other life-threatening problems increase as well [3].

In some cases, however, health problems can arise even without an increase in BMI [4]. Visceral abdominal adiposity, or central visceral obesity, is an independent risk factor for several significant comorbidities [5,6,7,8,9,10]. Approximately 52% of U.S. adults have abdominal obesity when defined using waist circumference [11], compared to the 42% when classified using BMI [12].

One approach to combatting the threat of obesity is a healthy diet. Daily energy balance is a function of energy intake and energy expenditure. Tipping the scale towards a negative energy balance, which is needed for weight loss, is best accomplished by decreasing total daily energy intake [13]. Thus, a healthy diet promotes the consumption of a variety of foods that help one stay within energy needs [14].

Milk has been purported to be part of a healthy diet because it is a rich source of nutrients, calcium, and protein. The relationship between milk consumption and weight regulation has been the topic of many recent publications [15,16,17,18]. Several meta-analyses conclude that milk and dairy consumption are correlated with decreased risk of obesity [19,20,21,22,23,24,25]. A few systematic reviews have found no difference [17,26,27], and one meta-analysis reports a positive relationship between milk intake and obesity [15].

When it comes to the effect of low-fat compared to high-fat milk on body weight, the literature appears mixed. A comprehensive review by Kratz et al. [28] determined that observational findings suggest an inverse association between high-fat dairy intake and obesity risk but remarked that the evidence was not conclusive. Of the 16 studies they reviewed, ten contrasted high-fat to low-fat dairy. All ten reported an inverse relationship between high-fat dairy consumption and obesity measures, while low-fat had either a positive correlation or no relationship. Only one of these investigations looked at milk specifically, and showed that skim and 1% milk, not whole, were positively associated with an increase of BMI in children. It appears that no randomized controlled trials have evaluated low- and high-fat milk consumption and their effects on obesity in adults.

Up to the present, few if any investigations have explored the influence of milk-fat on abdominal obesity as opposed to general obesity. A 24-week randomized controlled trial by Zemel et al. [29] found that dairy intake resulted in greater total fat loss and fat loss in the trunk region than a control group with normal calorie restriction, but the fat content of milk was not considered. A significant amount of visceral abdominal fat may pose greater risks than excess body weight and thus needs to be explored in conjunction with low- versus high-fat milk consumption [4,5,6,7,8,9,10].

The present study centered on determining the association between milk-fat consumption and obesity, particularly abdominal adiposity, in 13,544 randomly-selected U.S. adults. Additionally, the influence of age, gender, race, physical activity, leisure computer use and gaming, cigarette smoking, and alcohol use on the milk and obesity relationships were investigated.

## 2. Materials and Methods

### 2.1. Study Design

This investigation employed a cross-sectional design and used data from the National Health and Nutrition Examination Survey (NHANES), a government-run survey. To collect data, NHANES conducts comprehensive interviews and physical examinations on nationally representative samples.

Prior to data collection, each subject provided informed consent. Gathering of data was authorized by the Ethics Review Board for the National Center for Health Statistics and files were posted online for public use [30]. On the NHANES website, NHANES provides a full description of data collection methods and procedures [31]. This investigation utilized NHANES data collected from 2011–2016. The ethical approval code for NHANES data collection 2011–2016 is: Protocol #2011–17.

The current investigation consisted of a total of 13,544 participants, aged 20–79 years. Data collection was based on a four-stage random sampling of non-institutionalized U.S. adults. NHANES uses census data to select counties, blocks, dwelling units, and finally persons, selecting subsamples from each, in order for the data to be nationally representative [31]. In short, because participants were randomly selected, the results can be generalized to the U.S. adult population.

Adults were included in the study if they submitted written informed consent, participated in the necessary assessments, and were 20–79 years old at the time of data collection. They were excluded if their body mass index or sagittal abdominal diameter value was extreme (>3 standard deviations from the mean), were pregnant, reported irregular or varied milk intake, or they consumed only milks other than cow’s milk.

### 2.2. Instrumentation and Measurement Methods

#### 2.2.1. Milk Consumption

Milk consumption was measured using the NHANES Diet Behavior and Nutrition questionnaire [32]. Participants were questioned about their milk consumption frequency over the previous 30 days. Subjects were asked to include milk consumed on cereal or in hot cocoa made with milk. They were asked not to include small amounts added to other drinks or for cooking. For frequency of milk intake, participants answered: never; rarely (less than 1 time per week); sometimes (1–5 times per week); often (at least daily); or varied [32]. Participants who reported that their pattern of milk intake varied were excluded from the investigation (*n* = 47).

Participants were also divided by the type of milk they reported drinking: whole milk; 2% fat milk; 1%; non-fat or skim milk; soy milk; or other. Milk-fat percentage refers to the proportion of milk, by weight, that consists of butterfat. Regular milk drinkers were defined as persons that consumed any milk type at least five times a week. Participants who reported that they drank only soy milk (*n* = 82) or another type other than cow’s milk (*n* = 229) were not included in the analyses [32]. Dairy products other than liquid milk were not investigated in this study.

#### 2.2.2. Body Mass Index

Body mass index (BMI) is a common indicator of body weight that is not associated with height. It was one of the two outcome measures of the current study. Body mass index is determined by dividing weight (kg) by height squared (m^2^). Well-established categories are used to differentiate body weights among adults, including those with obesity [33]. Participants with extreme BMIs (>3 standard deviations above the mean: >50.6 kg/m^2^) were not included in the analyses (*n* = 184).

#### 2.2.3. Sagittal Abdominal Diameter

Sagittal abdominal diameter (SAD) was used as an index of visceral abdominal adiposity in the present study. SAD is an index of abdominal height [34]. It was measured with the subject in the supine position. Knees were bent at a 90-degree angle with hands resting on the chest and feet flat on the table. The NHANES examiner located the uppermost lateral border of the right and left ilium, making a mark on the abdomen along the iliac level line. An abdominal caliper was then used to measure the distance in centimeters between the small of the back and the abdominal mark. This measurement was taken at least two times to ensure accuracy. SAD has previously been shown to predict cardiometabolic disorders better than BMI and waist circumference [35,36,37], and correlates strongly with visceral fat measured by computed tomography [38].

### 2.3. Covariates

The latest NHANES definition of race includes six categories: Mexican American, Other Hispanic, White (single race), Black (single race), Asian (single race), or other, including multiracial persons. The six NHANES racial categories above were used in the present study.

Standing height was assessed using a stadiometer [34]. Weight was assessed in kilograms using a digital weight scale [34]. The only clothing worn by subjects was underwear, disposable shirt and pants, and slippers, which were provided to them [34].

Physical activity (PA) levels were measured via interview. As PA increases, energy expenditure increases [39]. Physically active people tend to weigh less and have less abdominal obesity than inactive people [40]. Hence, differences in PA were controlled statistically in this investigation. In the interviews, subjects were asked about the number of minutes spent in moderate and vigorous PA. Moderate PA was defined as activity that causes small increases in breathing or heart rate, such as carrying light loads or brisk walking. Vigorous PA was defined as activity that causes large increases in breathing or heart rate, such as lifting heavy loads or jogging [41].

Subjects were questioned on how many days per week they engaged in either type of activity, moderate or vigorous, as well as how much time was spent in activity on a typical day. For each intensity, days and minutes were multiplied together to calculate total minutes of moderate and total minutes of vigorous PA. These minutes were added together, resulting in total moderate and vigorous PA (MVPA).

Conversely, physical inactivity results in a reduction of total daily energy expenditure [39]. As such, it has been found to be a contributing factor for obesity and abdominal obesity in correlational and causative studies [42,43]. In today’s society, recreational computer use and gaming are often correlated with physical inactivity [44,45]. Therefore, subjects’ leisure computer use and gaming was measured via questionnaire in the current investigation. Specifically, information was gathered about recreational computer and gaming device usage, not associated with work or school, over the past 30 days. Subjects responded with no leisure computer use or gaming, less than one-hour of use per day, one hour, two hours, three hours, four hours, five or more hours, or do not know. Subjects who indicated more than zero and less than one-hour per day were assigned 0.5 h per day of use. The highest two categories were combined into one category, four hours or more per day.

Several studies in adults and children indicate that recreational computer use is a good predictor of overweight and obesity [46,47,48,49]. Moreover, concurrent validity was shown for the leisure computer use and gaming variable in the present study, given it was significantly and linearly related to BMI: (F = 15.9, *p* = 0.0002), and SAD: (F = 13.1, *p* = 0.0007), after adjusting for differences in age, gender, and race. Additionally, after controlling for all the covariates (age, gender, race, physical activity, alcohol habits, and cigarette use), leisure computer use and gaming remained significantly and linearly related to BMI (F = 18.5, *p* < 0.0001) and SAD (F = 15.7, *p* = 0.0003). Furthermore, in the present investigation, time spent in leisure computer use and gaming was linearly and inversely related to time spent in moderate physical activity, with age, gender, and race controlled (F = 4.8, *p* = 0.0330), and also after adjusting for all the other covariates (F = 5.5, *p* = 0.0232). Leisure computer use and gaming duration was also predictive of minutes of vigorous intensity physical activity per week, with age, gender and race controlled (F = 3.0, *p* = 0.0201), but not after adjusting for all the covariates (F = 2.1, *p* = 0.0862).

Cigarette smokers are less prone to have obesity than non-smokers, hence smoking was controlled statistically in this study [50,51,52]. Smoking was indexed using the average number of cigarettes smoked per day during the past 30 days [41].

Alcohol use was included as the final covariate in the current study. NHANES divides alcohol use into three categories, by gender. Women who had two or more alcoholic drinks a day or men who had three or more were considered heavy drinkers. Women who consumed >0 to <2 alcoholic drinks a day or men who consumed >0 to <3 drinks were considered moderate drinkers. Those who did not drink alcohol were categorized as abstainers. Alcohol use has been shown to be a risk factor for obesity [53].

### 2.4. Data Analysis

In the present investigation, strata, clusters, and individual sample weights were employed in the analyses so that the results can be generalized to all non-institutionalized adults in the United States. Although a sample size of 13,544 adults would usually result in substantial statistical power, NHANES’ use of a multi-level sampling strategy reduced power significantly. Specifically, instead of approximately 13,544 degrees of freedom (df), because of nesting, df in the denominator were defined as: clusters minus strata. This resulted in 47 df.

In the present investigation, the exposure variable was milk-fat consumption. There were two outcome measures: body mass index (BMI) and sagittal abdominal diameter (SAD), a measure of visceral abdominal adiposity. Age, sex, race, physical activity and inactivity levels, cigarette smoking, and alcohol use were included as covariates to control for their influence on the relationship between the exposure and outcome variables.

The two outcome variables, BMI and SAD, were treated as continuous variables. The exposure variable, the milk-fat content regularly consumed by participants, was treated as a categorical variable. One-way analysis of covariance (ANCOVA) was used employing the SAS SurveyReg procedure to determine mean BMI or SAD differences across the milk-fat categories. Potential mediating variables were controlled using partial correlation, and the least squares means (LSMeans) procedure was employed to generate adjusted means.

SAS version 9.4 (SAS Institute, Inc., Cary, NC, USA) was the software utilized to analyze the data. All of the analyses were two-sided and statistical significance was established as *p* < 0.05.

## 3. Results

There were 6743 men and 6801 women in the study (*n* = 13,544). Mean (±SE) age of the sample was 46.3 (±0.3) years. Table 1 displays the categorical variables of the study. Milk abstainers comprised 39.6% of the sample, 27.9% reported drinking milk sometimes, and 32.5% reported drinking milk often. The most commonly consumed milk was 2% milk-fat (25.8%), followed by full-fat milk (15.0%). For the covariate, leisure computer and gaming, computer use for school or work was not counted. A majority of subjects reported 0.5 hours (h) per week or less, and about 30% indicated they participated in 2 h or more per week. The percentage of the sample that were alcohol abstainers, moderate drinkers, or heavy drinkers was relatively equal.

The average (±SE) BMI of the subjects was 29.1 (±0.1) kg/m^2^, and the average sagittal abdominal diameter (SAD) was 22.7 (±0.1) cm. In the sample, 40% of subjects accumulated zero minutes of MVPA per week, with the mean (±SE) being 162 (±5) minutes per week. Additionally, for the less than 20% of the NHANES sample that smoked, average (±SE) number of cigarettes each month was 69.9 (±3.7).

Table 2 displays the percentiles associated with the continuous variables of the study. The median value for BMI was 28.0 (±0.1) kg/m^2^ and the median for SAD was 22.2 (±0.1) cm. Median minutes of MVPA per week was 56.6 (±7.4). The median number of cigarettes smoked over a 30-day period was 0 (±3.3) since most of the population were non-smokers.

Table 3 compares the BMI and SAD means according to milk-fat intake. Model 1 controlled for the demographic covariates only (age, gender and race). Milk abstainers and those who drank 1% or 2% had statistically equal BMIs, whereas subjects who drank non-fat or full-fat milk had significantly lower BMIs than the other groups (F = 4.5, *p* = 0.0038). The difference in BMI between non-fat or full-fat milk drinkers was not statistically significant.

In Table 3, Model 2 controlled for all seven of the covariates: age, gender, race, physical activity, leisure computer use and gaming, alcohol habits, and smoking. Similar to Model 1, statistically equal mean BMIs were seen in milk abstainers, those who drank 1%, and those who consumed 2% milk-fat. Drinking non-fat or full-fat milk compared to 1%, 2%, or abstaining from milk was associated with significantly lower BMIs (F = 4.1, *p* = 0.0061).

Milk-fat intake associated with SAD (i.e., abdominal adiposity) outcomes were different than for BMI. Specifically, the smallest average SAD was seen in non-fat milk drinkers. There was no difference in SAD between adults who consumed full-fat milk, 2%, 1%, or milk abstainers. However, adults who drank non-fat milk had significantly smaller sagittal abdominal diameters than each of the other milk-fat groups and abstainers (F = 5.4, *p* = 0.0011).

According to Model 2 in Table 3, with all the covariates controlled (age, gender, race, physical activity, leisure computer use and gaming, alcohol habits, and smoking), mean SAD was significantly smaller in non-fat milk drinkers compared to each of the other milk-fat groups: 1%, 2%, full-fat, and milk abstainers (F = 4.9, *p* = 0.0023). Although non-fat milk consumers had significantly smaller sagittal abdominal diameters, the other four milk-fat groups (those who abstained from consuming milk, those who consumed 1%, 2%, or full-fat milk) did not differ significantly from each other.

As displayed in Table 4, milk drinkers (regular consumers of skim milk, 1%, 2%, or full-fat milk) were compared to milk abstainers to determine the extent to which they differed in BMI and/or SAD. Results showed that with age, gender, and race controlled, milk-consumers did not differ from milk abstainers in BMI (F = 3.1, *p* = 0.0851). Similarly, regular milk-consumers and adults who abstained from drinking cow’s milk did not differ in their average SAD levels (F = 0.8, *p* = 0.3916). After adjusting for differences in all the potential confounders, mean differences remained statistically equal for BMI and SAD.

## 4. Discussion

The objective of this study was to assess the relationship between milk-fat intake and obesity. General obesity was measured using BMI and abdominal obesity, the primary focus of the study, was measured by SAD (sagittal abdominal diameter). The results were derived using a random sample of 13,544 U.S. adults as part of the National Health and Nutrition Examination Survey (NHANES). Another aim was to measure the magnitude of the influence of several demographic and lifestyle covariates on the association between milk-fat intake, obesity, and abdominal adiposity.

This study resulted in several meaningful findings. First, adults who regularly drank either non-fat or full-fat milk had significantly lower BMIs than adults who drank 1% or 2% milk or abstained from drinking milk. However, when the association between milk-fat intake and SAD was determined, only non-fat milk drinkers had significantly less abdominal adiposity compared to the other milk-fat groups. These results remained significant after adjusting for differences in the covariates. Additionally, when regular milk consumers were compared to abstainers, there were no differences in BMI or SAD. But when participants were divided based on milk-fat intake, there were significant differences.

Results of the present study support the U.S. Dietary Guidelines for Americans (2015–2020) [54], which recommend that adults drink low-fat milk, rather than high-fat milk. The guidelines define low-fat milk as non-fat milk or 1%. Given the lower prevalence of abdominal adiposity in non-fat milk consumers compared to adults who drink 1%, milk drinkers should probably consume non-fat milk, rather than 1%, even though both are labeled low-fat.

According to Table 1, a total of 19.6% of adults living in the United States drink low-fat milk. Only 10.4% drink non-fat milk. The percentage of adults who drink high-fat milk (2% and full-fat) is 40.8%, and 39.6% are not regular milk drinkers. It seems that many U.S. adults regularly drink high-fat milk, which is discouraged by the U.S. Dietary Guidelines [54] and is related to increased abdominal adiposity (SAD) in the current investigation.

Other studies have shown similar relationships between low-fat milk intake and BMI [55,56,57]. For example, an eight-week randomized crossover trial by Alonso et al. [58] compared the supplementation of either low-fat dairy or high-fat dairy to the foods eaten by young adults. They found that the inclusion of high-fat dairy significantly increased body weight compared to low-fat dairy.

Faghih et al. [59] randomized 100 premenopausal women to one of multiple protocols for eight weeks, all of which included a deficit of 500 kcal/day: (1) 500–600 mg/day dietary calcium, (2) 800 mg/day calcium supplement, (3) three servings/day of low-fat milk, or (4) three servings/day of soy-milk. Weight reductions in low-fat milk, soy milk, calcium supplement and control groups were 4.4 ± 1.9 kg, 3.5 ± 1.3 kg, 3.9 ± 2.4 kg and 2.9 ± 1.6 kg, respectively. The authors concluded that increasing low-fat milk intake reduced weight and waist circumference beyond an energy-restricted diet.

A true experiment (RCT) by Rossi et al. [60] treated 40 women with obesity for 12 weeks. Participants were given isocaloric diets, with one group consuming no dairy and the other consuming two servings of low-fat dairy per day. After 12 weeks, subjects in the no dairy group lost 6.0% body fat and subjects in the dairy group lost 10.8% body fat (*p* < 0.01). No significant difference was found in waist circumference or visceral fat in either group, beyond the effects from a low-calorie diet.

Conversely, there are some studies that support a relationship between high-fat milk intake and lower BMI. A correlational investigation by Crichton et al. [61] found high-fat milk consumption to be inversely related to BMI and waist circumference after having 1352 adults complete a food frequency questionnaire. Participants in the highest third of high-fat milk intake had significantly lower odds for general obesity and waist circumference, compared with those in the lowest third.

A cohort study by Holmberg et al. [62] studied 1589 Swedish men. Waist and hip circumferences were measured, and dietary analyses were completed before and after a 12-year project. The researchers determined that a high intake of high-fat milk was inversely related to risk of abdominal obesity (OR 0.52, 95% CI: 0.33–0.83) compared to a medium intake. Other studies have shown similar findings [28,63,64,65,66].

Investigations that have studied the association between low-fat milk consumption and abdominal obesity report results similar to the current investigation [67,68]. A cross-sectional study by Ardekani et al. [69] compared low-fat milk intake to high-fat milk intake in 8652 Iranian adults and found that adults who drank low-fat milk had significantly lower body fat percentages and abdominal obesity than the high-fat milk drinkers. Additionally, self-reported low-fat milk consumption was related to a lower waist-to-hip circumference in a study by Krachler et al. [70]. However, none of the above studies included SAD as a variable.

When combined, adults in the present study who regularly drank cow’s milk (i.e., skim milk, 1% milk-fat, 2%, or full-fat milk) did not differ from milk abstainers in BMI or sagittal abdominal diameter. However, there were significant differences in BMI and SAD when milk-fat was accounted for, especially when abdominal adiposity was the outcome of interest. These findings seem to support the notion that milk-fat may play a role in visceral abdominal fat differences.

The effect of general milk consumption, regardless of milk-fat content, on body weight and obesity has been studied many times, especially in children and adolescents. In a recent review of the role of milk in the development of obesity in children, researchers found mixed results, mostly indicating no relationship or an inverse association, with a minority of studies showing a positive correlation [71]. Additionally, in a study of over 1200 adolescents from Portugal, ages 15–18, milk intake in general was associated with lower levels of abdominal obesity [72]. On the other hand, in a prospective study of almost 100,000 Danish adults, consumption of 1–3 glasses of milk per week was associated with a 12% greater risk of developing overweight or obesity compared to milk-abstainers, and milk drinkers, in general, had a 6% higher risk compared to abstainers [73]. In this Danish study, high-fat milk drinkers had the same risk of becoming overweight or obese as milk abstainers [73].

The relationships between milk-fat intake and BMI, and milk-fat intake and SAD differed in the present study. Differences might have occurred because BMI is a general or gross index of body weight for a given height, whereas SAD is a sensitive measure of adiposity, particularly visceral abdominal fat. BMI does not differentiate between lean mass and body fat. However, SAD is a very good index of visceral abdominal fat when compared to computed tomography [38]. Furthermore, SAD correlates with increased risk of disease [4,5,6,7,8,9,10], even if general obesity is not present. Elevated levels of visceral fat seem to pose a greater risk than excess body weight alone [56,74,75]. Consequently, SAD provides more valuable information than BMI.

Reverse causation could account for some of the results indicating that adults who drink high-fat milk have lower BMIs. Specifically, instead of milk-fat influencing obesity, body weight could affect the type of milk selected by individuals. Adults with lower BMIs might drink full-fat milk because they like the creamier taste compared to fat-free milk, or they might feel that they need the extra calories. These adults might not be concerned about the extra calories in whole milk because they are lean.

Several potential mechanisms could account for the inverse association found between non-fat milk intake and SAD in the current study. High-fat milk has a higher caloric content compared to low-fat milk. A diet with an excess of calories is known to increase fat stores in the body [13]. Additionally, individuals often do not compensate for the consumption of liquid calories, which could result in excess intake of energy [76,77]. Similar findings have been reported in children [78]. Moreover, it is possible that adults who regularly drink low-fat milk are more health conscious in other areas of their diet and consequently select foods that are lower in calories compared to high-fat milk drinkers. There could exist other variances in subjects’ lifestyles, not including physical activity, smoking, and alcohol use, which were controlled in the present study.

The current study had several limitations. First, conclusions about the present study must be made carefully because it was a cross-sectional investigation. Cross-sectional studies cannot control for all possible confounding factors; thus, causation cannot be concluded. Statistical adjustments were made for differences in demographic and lifestyle variables at the analysis level of this study. Additionally, information about lactose intolerance was not collected by NHANES, so differences could not be evaluated or controlled. Similarly, medication use and dietary intake were not controlled. Moreover, leisure computer use and gaming was employed as a single variable to represent physical inactivity. Although this covariate was predictive of higher levels of BMI and SAD, and inversely related to physical activity, there are many other sedentary activities other than computer use and gaming that were not measured. Furthermore, some individuals are sedentary long hours each day, regardless of their leisure computer use and gaming habits, whereas others are physically active during the day and engage in computer use and gaming only during leisure hours. Finally, consumption of milk and milk-fat were assessed using a questionnaire, so some error associated with self-reported information is possible.

The present study also had several strengths. First, multiple races and a large, randomly selected sample (*n* = 13,544) were included in the study. This makes the findings generalizable to the U.S. noninstitutionalized, civilian adult population. Second, SAD was used as a measure of visceral abdominal adiposity, which is not a usual measurement in the literature but correlates strongly with visceral adiposity. Third, the relationship between low-fat milk consumption and lower levels of SAD was consistent and highly significant. Fourth, several potential confounding factors were controlled statistically, reducing their impact on the relationship between milk-fat intake, BMI, and SAD. Fifth, this study sheds light on the differences between low-fat and high-fat milk intake in relation to visceral abdominal adiposity, particularly SAD, which is not common among milk and dairy investigations.

Overall, the present investigation warrants further study in the area of milk-fat and obesity, particularly abdominal obesity. Although long-term randomized controlled trials are ideal, compliance is typically a problem. Hence, prospective cohort studies and cross-sectional investigations that control for more potential confounders are warranted. Moreover, the effect of milk-fat differences on more precise measures of body composition, such as DXA, would be valuable.

## 5. Conclusions

The literature surrounding milk-fat intake and obesity in adults is mixed and the topic is controversial. In the current study, both overall obesity and abdominal adiposity were assessed. Sagittal abdominal diameter (SAD) is an excellent index of visceral abdominal adiposity, whereas BMI is a general index of body weight for height. SAD also seems to be a better forecaster of morbidity risk than BMI. Both non-fat and full-fat milk intake were associated with lower BMI levels. However, only non-fat milk consumption was related to a lower SAD. High-fat milk intake was not. The present investigation highlights the potential advantage tied to U.S. adults drinking low-fat milk. The findings support current U.S. guidelines which urge the use of low-fat milk as part of a nutritious diet.

## Figures and Tables

**Table 1:** Information about the sample (*n* = 13,544).

Variable	*n*	Weighted %	SE
Gender			
Men	6743	49.7	0.4
Women	6801	50.3	0.4
Race			
Mexican American	1957	9.0	1.2
Other Hispanic	1497	6.3	0.8
White (single race)	4893	65.1	2.2
Black (single race)	3060	11.2	1.2
Asian (single race)	1703	5.4	0.6
Other or Multi-Racial	434	3.0	0.3
Leisure computer use			
0 h/week	4134	22.4	1.0
0.5 h/week	3283	28.3	0.7
1 h/week	2259	19.6	0.6
2 h/week	1736	13.9	0.4
3 h/week	815	6.1	0.3
4 or more	1314	9.7	0.4
Alcohol Use			
Abstainer	5089	30.3	0.9
Moderate Drinker	4047	33.3	0.9
Heavy Drinker	4408	36.4	0.7
Milk Intake Frequency			
Never/Rarely	5547	39.6	0.8
Sometimes	3803	27.9	0.6
Often	4194	32.5	0.7
Milk-fat Consumed			
Full-Fat	2435	15.0	1.4
2% Milk	3588	25.8	1.3
1% Milk	1025	9.2	1.1
Non-fat Milk	949	10.4	1.2
Milk Abstainers	5547	39.6	0.7

The Weighted % column shows the distribution of subjects after the NHANES sample weights were applied. The Weighted % values are more meaningful than the number of subjects because they consider the sample weights and reflect the percentage of the U.S. population that practice the behavior. SE is the weighted percentage standard error.

**Table 2:** Percentiles for the continuous variables.

Variable	Percentile (±SE)				
	5th	25th	50th	75th	95th
Body Mass Index (kg/m^2^)	20.6 ± 0.1	24.4 ± 0.1	28.0 ± 0.1	32.6 ± 0.2	41.4 ± 0.3
Sagittal Diameter (cm)	16.5 ± 0.1	19.4 ± 0.1	22.2 ± 0.1	25.5 ± 0.1	30.5 ± 0.2
Smoking (cigarettes/30 days)	0 ± 3.3	0 ± 3.3	0 ± 3.3	0 ± 3.3	582.5 ± 36.0
MVPA (minutes/week)	0 ± 8.3	0 ± 8.3	56.6 ± 7.4	236.4 ± 8.2	680.7 ± 27.6

SE: standard error. MVPA: minutes of moderate and vigorous physical activity combined. Less than 20% of the sample smoked, hence zero is reported until the 95th percentile. Similarly, adults in the 5th and 25th percentiles for MVPA accumulated less than 10 min of MVPA per week.

**Table 3:** Differences in mean (±SE) BMI or sagittal abdominal diameter by milk-fat category in randomly selected U.S. adults, after controlling for differences in potential confounders.

	Milk-Fat Content Typically Consumed						
	Milk Abstainer	Full-Fat	2%	1%	Non-Fat
Model:	Mean ± SE	Mean ± SE	Mean ± SE	Mean ± SE	Mean ± SE	F	*p*
Model 1							
BMI	29.1 ± 0.1 ^b^	28.2 ± 0.3 ^a^	29.1 ± 0.2 ^b^	29.2 ± 0.3 ^b^	28.1 ± 0.4 _a_	4.5	0.0038
SAD	22.6 ± 0.1 ^b^	22.4 ± 0.2 ^b^	22.8 ± 0.2 ^b^	22.7 ± 0.2 ^b^	21.7 ± 0.2 a	5.4	0.0011
Model 2							
BMI	29.0 ± 0.1 ^b^	28.2 ± 0.3 ^a^	29.0 ± 0.2 ^b^	29.1 ± 0.3 ^b^	28.1 ± 0.4 ^a^	4.1	0.0061
SAD	22.6 ± 0.1 ^b^	22.4 ± 0.2 ^b^	22.8 ± 0.2 ^b^	22.7 ± 0.2 ^b^	21.8 ± 0.2 ^a^	4.9	0.0023

^a,b^ Means on the same row with the same superscript letter are not statistically different (*p* > 0.05). BMI: body mass index. SAD: Sagittal abdominal diameter. The distribution of subjects was Milk Abstainers: *n* = 5541, 57.0%; Full-fat: *n* = 1219, 12.5%; 2%: *n* = 1857, 19.1%; 1%: *n* = 540, 5.6%; and Non-fat: *n* = 571, 5.9%. When summed, percentages may not equal 100% because of rounding. Participants who reported drinking milk “sometimes” were not included in the analysis. SE is standard error of the mean. Model 1 compares BMI and SAD means separately, after controlling for age, gender, and race. Model 2 compares BMI and SAD means separately, after adjusting for age, gender, race, physical activity, leisure computer use and gaming, alcohol habits, and cigarette use. The NHANES sample weights assigned to each subject are not reflected in the number of participants in each category (*n*). However, the sample weight is applied to the percentage (%) following the sample size. Therefore, the percentage is a more meaningful value.

**Table 4:** Mean (±SE) differences in BMI or sagittal abdominal diameter between milk consumers and milk abstainers, after controlling for differences in potential confounders.

	Milk Consumers	Milk Abstainers		
Model:	Mean ± SE	Mean ± SE	F	*p*
Model 1				
BMI	28.7 ± 0.2	29.1 ± 0.1	3.1	0.0851
SAD	22.5 ± 0.1	22.7 ± 0.1	0.8	0.3916
Model 2				
BMI	28.7 ± 0.2	29.0 ± 0.1	3.7	0.0609
SAD	22.5 ± 0.1	22.6 ± 0.1	0.5	0.4712

Milk consumers included milk drinkers who reported consuming full-fat, 2%, 1%, or skim milk often. Abstainers were adults who reported milk consumption less than once per week. The distribution of subjects was: Milk Consumers: *n* = 4194 and Milk Abstainers: *n* = 5547. Participants who reported drinking milk “sometimes” were not included in the analysis. SE is standard error of the mean. Model 1 compares BMI and SAD means separately, after controlling for age, gender, and race. Model 2 compares BMI and SAD means separately, after adjusting for age, gender, race, physical activity, leisure computer use and gaming, alcohol habits, and cigarette use.

## Data Availability

All data supporting reported results can be found online as part of the National Health and Nutrition Examination Survey (NHANES). The data are free and can be found at the following website: https://wwwn.cdc.gov/nchs/nhanes/Default.aspx (accessed on 25 May 2021).
